# Subcutaneous tissue reaction and gene expression of inflammatory markers after Biodentine and MTA implantation

**DOI:** 10.1590/0103-6440202203562

**Published:** 2022-03-07

**Authors:** Raquel Assed Bezerra Silva, Patrícia Gaton-Hernandez, Carolina Maschietto Pucinelli, Francisco Wanderley Garcia de Paula e Silva, Marília Pacífico Lucisano, Alberto Consolaro, Rafaela Cardoso de Sá, Lisa Danielly Curcino Araujo, Manoel Damião Sousa-Neto, Léa Assed Bezerra Silva

**Affiliations:** 1 Department of Pediatric Dentistry, School of Dentistry of Ribeirão Preto, University of Sao Paulo, São Paulo, SP, Brazil; 2 Department of Integrated Paediatric Dentistry, School of Dentistry, University of Barcelona, Barcelona, Spain; 3 Department of Oral Pathology, Bauru Dental School, University of Sao Paulo, São Paulo, SP, Brazil.; 4 Department of Restorative Dentistry, School of Dentistry of Ribeirão Preto, University of Sao Paulo, São Paulo, SP, Brazil.

**Keywords:** Pro-Root MTA, Biodentine, Immune-inflammatory response, Subcutaneous connective tissue, Cytokines

## Abstract

The aim of this study was to evaluate the subcutaneous connective tissue response of isogenic mice exposed to tricalcium silicate (Biodentine) and aggregated mineral trioxide (ProRoot MTA). A total of 120 mice were divided into 4 groups in 3 different experimental periods (7, 21 and 63 days): Biodentine; Pro-Root MTA; zinc oxide-eugenol and; Negative control - Sham. After the experimental periods microscopic descriptive, semi-quantitative and quantitative analysis of the inflammatory process were analyzed on H&E sections and evaluation of the gene expression of *Il10*, *Infg*, *Il6*, *Il1r1* and *Tnf* (qRT-PCR) were performed. The data obtained were analyzed using the chi-square test and two-way analysis of variance (ANOVA) followed by the Bonferroni post-test (5% significance level). *Results:* In the microscopic analysis, a slight inflammatory infiltrate was observed, with a predominance of sparse macrophages and polymorphonuclear cells, slight tissue fibrosis, regular fibrous capsule and with dystrophic calcifications, in all groups that received the materials (Biodentine and Pro-Root MTA). In parallel, all materials modulated the gene expression of the different cytokines and receptors evaluated. *Conclusion:* Pro-Root MTA and Biodentine showed a tissue compatibility, mediated inflammation, with increased fibrous tissue and production of pro- and anti-inflammatory cytokines.

## Resumo

Objetivo: Avaliar a resposta do tecido conjuntivo subcutâneo de camundongos isogênicos expostos à Biodentine™ e ao Trióxido Mineral Agregado (MTA). Métodos: Um total de 120 camundongos foram divididos em 4 grupos e 3 períodos experimentais diferentes (7, 21 e 63 dias): Biodentine™ (Septodont, Saint Maur des Fosses, França); Pro-Root MTA (Dentsplay, Tulsa, EUA); óxido de zinco eugenol (Biodinâmica Química e Farmacêutica LTDA., Ibiporã, PR - Brasil); e controle negativo - Sham. Após os períodos experimentais, análises microscópicas descritivas, semiquantitativas e quantitativas do processo inflamatório foram analisadas nos cortes de H&E e ainda, foi realizada a avaliação da expressão gênica de *Il10*, *Infg*, *Il6*, *Il1r1* e *Tnf* (qRT-PCR). Os dados obtidos foram analisados por meio do teste do qui-quadrado e da análise de variância (ANOVA) two-way, seguido do pós-teste de Bonferroni (nível de significância de 5%). Resultados: Na análise microscópica observou-se discreto infiltrado inflamatório, com predomínio de macrófagos esparsos e polimorfonucleares, leve fibrose tecidual, cápsula fibrosa regular e com calcificações distróficas, em todos os grupos que receberam os materiais (Biodentine ™ e Pro-Root MTA). Paralelamente, todos os materiais modulam a expressão gênica das diferentes citocinas e receptores avaliados. Conclusão: Pro-Root MTA e Biodentine™ mostraram compatibilidade tecidual, inflamação mediada, com aumento do tecido fibroso e produção de citocinas pró- e antiinflamatórias.

## Introduction

Pulpotomy is a conservative endodontic procedure indicated for teeth that have extensive caries lesions, but without evidence of infection in the radicular pulp tissue, traumatic pulp exposure, in addition to the absence of spontaneous and persistent pain, abscess and fistula ^1^. The success rate of this treatment, when correctly indicated, varies between 90 to 100% ^2^.

The ideal biological response expected after pulpotomy is the formation of a mineralized tissue barrier over the remaining radicular pulp, protecting this tissue from additional irritating stimuli. This biological process depends on the correct execution of all operative phases, as well as the choice of a biological protective material, which presents tissue compatibility and repair capacity.

Mineral trioxide aggregate (MTA) has been recognized as the gold-standard material for conservative pulp vitality treatments, with high success rates (90%-100%) in clinical, radiographic, and histopathologic studies ^3^ ^,^ ^4^ ^,^ ^5^. The effect of MTA on pulpal tissue has certain similarities to that produced by calcium hydroxide. Its high pH generates a narrow zone of coagulation necrosis that is an initiator of a wound healing response. Next to that zone, a reparative dentin is formed. In addition to tissue compatibility and antibacterial properties, MTA induces a release of wound healing signals (growth factors) from dentin and promotes a very tight seal with the dentin walls due to a layer of hydroxyapatite created as a link, forming a physical and chemical bond between MTA and dentin ^6^. However, MTA has some disadvantages, such as long hardening time (2.75 hours), need for hydration during this period ^7^, and handling difficulties ^8^. Besides that, some brands offer a MTA with grey color, favoring a dental color change.

Currently, the search for biological materials has grown in Dentistry, in an attempt to promote an adequate repair process, without cytotoxic or irritating action to the organism. Biodentine™ (Septodont - St-Maur-des-Fossés - France), a cement based on tricalcium silicate, was introduced on the market and has shown satisfactory results in clinical and laboratory researches ^9^, being able to stimulate the formation of tertiary dentin on the exposed pulp tissue ^10^. The main advantages of using Biodentine™ over MTA include ease of handling, high viscosity, shorter hardening time and better physical properties ^11^, besides presenting color stability ^12^.

Application of Biodentine on vital pulp tissue stimulates early formation of reparative dentin, resulting in complete dentin bridge, absence of an inflammatory pulp response and layers of well-arranged odontoblast-like cells ^11^. The interactions of Biodentine dental tissues lead to a marginal sealing, with bond strength to dentine compared to MTA, due to penetration of Biodentine into the dentin tubules forming tag-like structures and promoting a micromechanical retention ^13^. There is evidence for the positive effects of Biodentine on vital pulp cells due to its high biocompatibility, antibacterial properties and excellent bioactivity. However, the literature is still inconclusive in choosing the most reliable pulp capping material, highlighting the relevance of understanding the mechanisms that induce repair and mineralization of both materials. Although there are studies in the literature evaluating MTA and Biodentine™ ^14^, the study of the immune-inflammatory response is important, including the detection of pro and anti-inflammatory markers stimulated by these materials, to elucidate the mechanisms involved in the repair process and to support the clinical use.

Would healing is a complex process involving different cellular, molecular and biochemical events. Briefly, healing response can be divided into three distinct but overlapping phases, being them inflammation, proliferation and maturation. Among the inflammatory reactions, local release of cytokines and growth factors plays important functions during the repair process. These molecules may have more than one specific effect on cells, depending on the local conditions. In addition, the responses are also mediated by cell surface receptors ^15^.

Tumor necrosis factor-α (TNF-α), interleukin-1 (IL-1) and IL-6 are proinflammatory cytokines. TNF-α and IL-1 may lead to both activation of resident stromal and immune cells and recruitment of leukocytes, mostly neutrophils, from circulation with subsequent activation of phagocytosis and secretion of antimicrobial peptides. IL-6 was described as a mediator of inflammation and lymphocyte differentiation ^16^. Signaling pathway of IL-1 is associated with activation of a cascade characteristic of innate immunity Toll-like receptors, via the receptor complex IL-1R1.

Interferon-γ (IFN-γ) is considered a major mediator of macrophage inflammation, by inducing a dramatic increase in the production of inflammatory mediators ^17^. On the other hand, principal actions of IL-10 are primarily considered anti-inflammatory and inhibitory, targeting both innate and adaptive immune responses and playing immunosuppressive functions to reduce tissue damage caused by excess and uncontrolled inflammatory responses ^18^ ^,^ ^19^.

Considering that wound repair is characterized by a well-orchestrated response, studying the behavior of the involved molecules, after biomaterials exposure, presents scientific and clinical relevance. 

Thus, the objective of this study was to evaluate the tissue response, immune response and participation of pro- and anti-inflammatory cytokines induced by Biodentine™ and MTA materials in subcutaneous connective tissue of isogenic mice. The null hypothesis was that immuno-inflammatory reactions would not be different between MTA and Biodentine.

## Material and methods

The design of the study was based on the ARRIVE (*Animal Research: Reporting of In Vivo Experiments*) guidelines. Initially, the project was submitted and approved by the Ethics Committee on the Use of Animals of the Faculty of Dentistry of Ribeirão Preto, University of São Paulo (CEUA / FORP-USP) (Process Number 2015.1.890.58.4). Care for the welfare of animals followed the ethical standards and principles adopted by CEUA / FORP-USP and the Normative Resolutions of the National Council for the Control of Animal Experimentation (CONCEA), regulated by the Brazilian Federal Constitution. Tests were conducted as determined by ISO 10993-6: 2007.

## Materials Preparation

The materials Biodentine™ (Septodont, Saint Maur des Fosses, France), Pro-Root MTA (Dentsply, Tulsa, USA) and zinc oxide-eugenol (Biodinâmica Química e Farmacêutica LTDA., Ibiporã, PR - Brasil) were handled according to their respective manufacturers in laminar flow for the maintenance of the aseptic chain. Thirty specimens of each material were prepared using as model a Teflon matrix (5 mm in height and 1.5 mm in diameter). The composition and manufacturer information of the materials are listed in Table 1.

**Table 1:** The composition and manufacturers of dental materials

Product/manufacturer	Composition
ProRoot MTA/ Dentsplay, Tulsa, USA	Tricalcium silicate (66.1%), dicalcium silicate (8.4%), tricalcium aluminate (2.0%), tetracalcium aluminoferrite, calcium sulphate bismuth oxide (14%), calcium oxide (8%), silicon oxide (0.5%), and aluminium oxide (1.0%)
Biodentine®/ Septodont, St. Maur des Fosses, FRANCE	Powder: tricalcium silicate (80.1%), dicalcium silicate, calcium carbonate (14.9%), iron oxide, and zirconium oxide (5%). Liquid: water, calcium chloride, and partially modified polycarboxylate
Zinc oxide-eugenol/ Biodinâmica Química e Farmacêutica LTDA., Ibiporã, PR - Brasil	Powder: Zinc Oxide Liquid: Eugenol, glacial acetic acid

### Obtaining the Animals

A total of 120 isogenic male mice of the BALB/c strain, from 6 to 8 weeks of age, weighing an average of 20 grams, were purchased from the central animal facility of the University of São Paulo, Ribeirão Preto, Brazil (USP). All animals were kept at the animal facility of the School of Dentistry of Ribeirão Preto - University of São Paulo, in polypropylene cages, with constant temperature (22 ± 2ºC) and relative humidity (55 ± 10%), in a 12:12 hour dark light cycle, with standard food and free access to water.

### In vivo study of subcutaneous tissue reaction in mice

After a week of acclimation, the animals were anesthetized with intramuscular injection of ketamine 10% (Agener União Química Farmacêutica Nacional S/A, Embu-Guaçu, SP, Brasil) and 2% xylazine (Dopaser, Laboratorios Calier, SA, Barcelona, Espanha) in the proportion of 0.2 mL/kg and 0.8 mL/kg, respectively, immediately before the surgery. Then, trichotomy on the animal's back and antisepsis of the region with 1% chlorhexidine digluconate was performed. The incision was made with sterilized surgical scissors in the dorsal region, with a size of 1 cm, followed by divulsion. After positioning the specimen inside the tissue, the skin was sutured using silk suture (Vicryl 4-0, Ethicon, Johnson & Johnson). The animals were kept at animals’ facility at School of Dentistry of Ribeirão Preto - USP, during the experimental periods, with food and water *ad libitum*.

The experimental groups were divided according to the material inserted in the subcuaneous tissue of each animal. The distribution of groups, materials used, number of animals and experimental periods are shown in Table 2.

**Table 2:** Distribution of experimental groups, materials used, number of animals and experimental periods

Group	Material	Animals per period	Experimental Periods
Experimental	Biodentine™	n=10	7, 21 e 63 days
Experimental	MTA	n=10	7, 21 e 63 days
Experimental	Zinc oxide-eugenol	n=10	7, 21 e 63 days
Negative control	Surgical procedure only (*sham*)	n=10	7, 21 e 63 days

At the end of each experimental period (7, 21 and 63 days), the animals were euthanized by anesthetic overdose. The connective tissue block containing the specimen with the material was removed with surgical scissors and divided into two parts. The upper part, the tissue was removed, fixed in 10% formaldehyde and sent for routine histotechnical processing for further microscopic analysis of H&E stained sections. The lower part of the connective tissue block containing the specimen with the material was immersed in an RNAlater^®^ solution (Ambion^TM^, Carlsbad, CA, USA) for further analysis of gene expression using the qRT-PCR technique.

### Histotechnical processing and microscopic evaluation

The set containing the specimen of the tested material, the surrounding portion of the subcutaneous connective tissue and the skin were removed with sterile surgical scissors, fixed by immersion in 10% buffered formalin for 24 hours at room temperature and then washed for approximately 4 hours in running water. Using a needle, the specimens were gently removed from the subcutaneous tissue. Then, the pieces were subjected to routine histotechnical processing, as described in the study by Pucinelli *et al.*, ^20^ and included in paraffin.

Semi-serial sections of 5μm (10 to 15 slides with 2 cuts per slide), with intervals of 15μm, were obtained over the entire tissue. Then, the sections were stained with HE, and submitted to analysis under a conventional optical microscope Axio Imager.M1 (Carl Zeiss MicroImaging GmbH, Göttingen, Alemanha), with attached AxioCam MRc5 camera (Carl Zeiss MicroImaging GmbH, Göttingen, Germany). All analyzes were performed by a single experienced evaluator, without previous knowledge of the group to be analyzed. Three regions of each section were evaluated and the average field was always the chosen field.

### Descriptive microscopic analysis

A qualitative description of the histopathological events observed in the reaction tissue in contact with the tested materials or in the control group (Sham) was performed in each experimental period (7, 21 and 63 days).

### Semi-quantitative microscopic analysis

In the tissue peripheral to the tested material, phenomena related to the fibrous and inflammatory infiltrate of the tissue adjacent to the evaluated materials were observed, according to the following criteria described by our research group previously ^20^:

- Collagen Fiber Formation: The number and density of collagen fibers in the reaction tissue surrounding the evaluated materials were analyzed and the score description is described in Table 3.
- Inflammatory infiltrate: The density of polymorphonuclear (PMN) surrounding the reactional tissue adjacent to the evaluated materials was analyzed and the score description is described in Table 4.

**Table 3:** Collagen Fiber Formation scores description

Analysis	Scores	Fibrosis Classification	Description
Collagen Fiber Formation	0	Absent	Absence of collagen fiber formation
	1	Mild	The collagen fibers were individualized just like normal connective tissue, interspersed with negative spaces indicative of non-fibrous components of extracellular matrix
	2	Moderate	Some areas the collagen fibers were individualized, but with alternating areas of eosinophilic extracellular matrix without linear and undulate formations
	3	Intense	The collagen fibers are present in the middle of an extracellular eosinophilic matrix, without typical linear and undulate formations, not allowing its individual observation

**Table 4:** Inflammatory infiltrate score description

Analysis	Scores	Fibrosis Classification	Description
Inflammatory infiltrate	0	Absent	Absence of inflammatory infiltrate
	1	Mild	1 to 10 PMN were observed in the reactional tissue
	2	Moderate	11 to 20 PMN were observed in the reactional tissue
	3	Intense	More than 21 PMN were observed in the reactional tissue.

PMN: polymorphonuclear

### Quantitative microscopic analysis

The Figure 1 is a representative image describing the mesurament of the fibrous capsule thickness. It was measured in images obtained from 3 regions of each section, using 3 sections per specimen, in a 10 × magnification. The software used was AxioVision Rel, v 4.8, Carl Zeiss MicroImaging GmbH using the “measures” tool. The results were expressed in µm.

**Figure 1:**
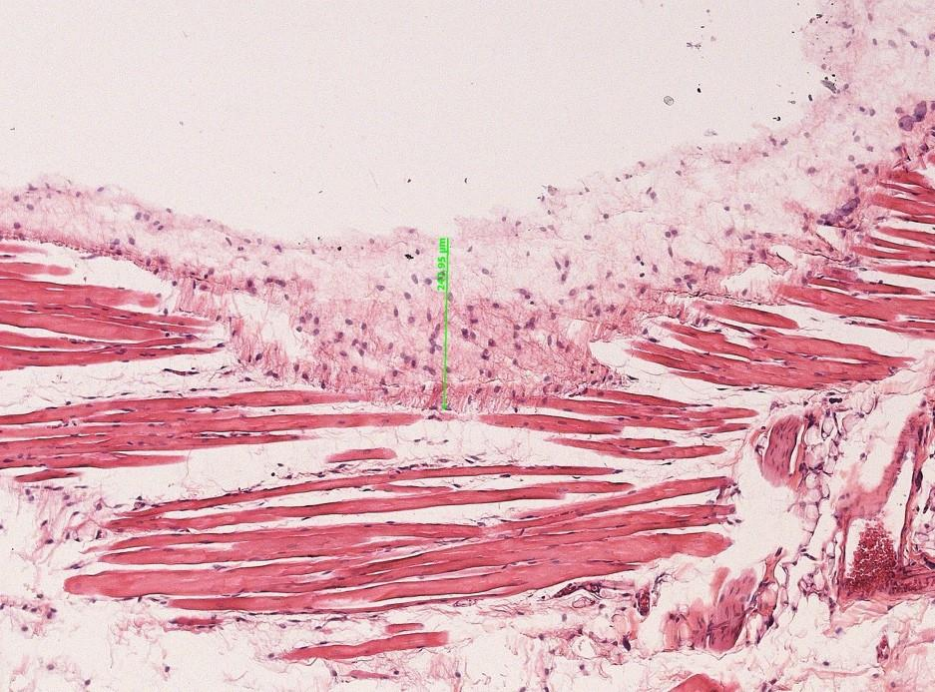
Representative image of the measurement of the fibrous capsule thickness in the subcutaneous tissue of mice, after implantation with Biodentine™, MTA, Zinc oxide-eugenol and from the Sham group (10 × magnification).

Evaluation of gene expression - qRT-PCR (Real Time Polymerase Chain Reaction)

To perform the qRT-PCR analysis, the pieces carrying the lower part of the set containing the piece of the tested material and the surrounding portion of the subcutaneous connective tissue were used. The pieces were removed and dipped in a solution of RNAlater^®^ (AmbionTM, Carlsbad, CA, EUA) for subsequent performance of qRT-PCR to detect messenger RNA for cytokines *Il10, Infg, Il6, Tnf* and receptor *Il1r1*. Total RNA was extracted using the column method, using a method based on guanidine thiocyanate (RNEasy, Qiagen Inc.). Then, reverse transcription was performed for the synthesis of complementary DNA (cDNA) and, finally, the polymerase chain reaction. *Primers* and probes for *Il10* (Mm00439614), *Infg* (Mm01168134), *Il*6 (Mm00446190), *Il1r1* (Mm00434237) and *Tnf* (Mm00443258) were obtained commercially and are private properties, therefore the nucleotide sequences are not available (TaqMan® Gene Expression Assay, Applied Biosystems, Foster City, CA, USA). The glyceraldehyde-3-phosphate dehydrogenase gene (Gapdh; Hs 138400) was used as a reference. The qRT-PCR reactions were performed in duplicate, using the StepOne Plus (Applied Biosystems) device.

Amplification was carried out under the following conditions: activation of the AmpliTaq Gold Enzyme polymerase at 95°C for 2 minutes, followed by 40 cycles of 95°C for 1 second for DNA denaturation and 60°C for 15 seconds for *primer* annealing and polymerization. The results were analyzed based on the value of the threshold cycle (Ct, *cicle threshold*). As a negative control, deionized distilled water was used, subjected to the reaction with each pair of *primer* and *probe* sequences used. For each gene, the calculation of the relative expression was performed from the difference between the ∆Ct of the samples and the ∆Ct presented by the control (ΔΔCt), based on the equation: *Relative expression = 2* ^*-∆∆Ct*^ *.*

### Statistical analysis

Semi-quantitative data were compared using the Kruskal-Wallis test and the Dunn post-test. Quantitative data were analyzed using two-way analysis of variance (ANOVA) followed by the Bonferroni post-test. The level of significance adopted was 5%. All analyzes and graphic representations were performed using the GraphPad Prism 7 Software (GraphPad Software Inc., San Diego, CA, EUA).

## Results

### Descriptive Microscopic Analysis

###  Biodentine ™ 

Figure 2 is a representative image of the tissue response observed in the subcutaneous conjunctive tissue at 7 (A, B), 21 (C, D) and 63 (E, F) days after implantation of the Biodentine™ cylinder. In the 7-day experimental period, the formation of a disorganized fibrous membrane and great infiltration of polymorphonuclear and macrophages are observed. In this area, either focally or just below the reaction tissue, basophilic degeneration of the tissue was noted, characterized by the loss of cell boundaries with purple coloration of the intercellular spaces. Isolated points of dystrophic calcification were also observed.

**Figure 1:**
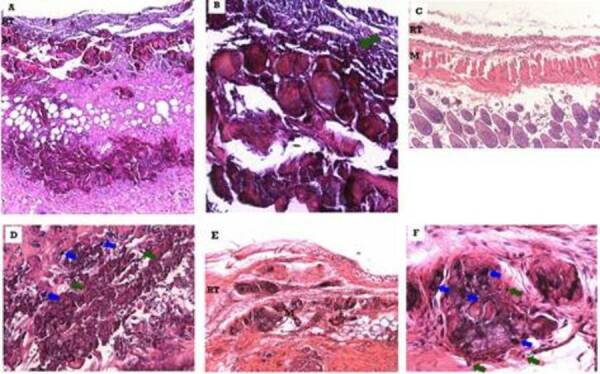
Representative image of the measurement of the fibrous capsule thickness in the subcutaneous tissue of mice, after implantation with Biodentine™, MTA, Zinc oxide-eugenol and from the Sham group (10 × magnification).

At 21 days, at the interface around the material, the reaction tissue showed a degree of slight fibrous and mixed inflammatory infiltrate composed of macrophages and polymorphonuclear cells, ranging from mild to moderate. The thickness of the reaction tissue was uniform and the organizational structure was rudimentary. In the most peripheral part of the material, the connective tissue showed many basophilic formations, indicating the presence of dystrophic calcifications.

At 63 days, the reactional tissue presented with slight fibrous and mild mononuclear inflammatory infiltrate, predominantly macrophage and with eventual randomly dispersed polymorphonuclear cells. The thickness of the capsule formed was uniform. Many dystrophic calcifications have been found on the periphery of the reaction tissue.

###  Pro-Root MTA 

Figure 3 shows the tissue response in the subcutaneous conjunctive tissue at 7 (A, B), 21 (C, D) and 63 (E, F) days after implantation of the Pro-Root MTA cylinder. At 7 days, a uniform tissue reaction was observed, with a slight degree of fibrous. The intensity of the infiltrate varied, in some specimens, from moderate to intense and, in others, from mild to moderate. Despite the macrophage predominance, polymorphonuclear cells were common. In two specimens, particles of material surrounded by the reaction tissue were observed.

**Figure 2:**
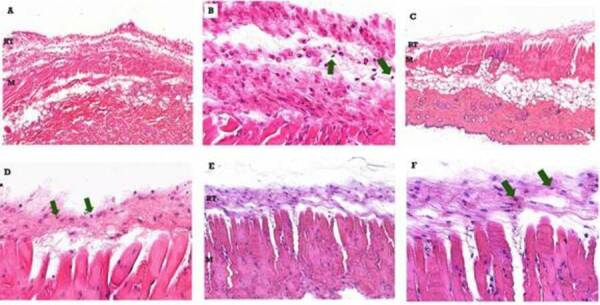
Representative photomicrographs of the tissue response observed in the subcutaneous connective tissue of mice after implantation of the Biodentine™ cylinder (10 × and 40 x magnifications) Blue arrow - PMN and Green arrow - macrophages. M - muscle, RT - reactional tissue. (A) - After 7 days were observed a disorganized fibrous membrane. Also, focally or just below the reaction tissue, basophilic degeneration of the tissue was noted, characterized by the loss of cell boundaries with purple coloration of the intercellular spaces. (B) - Detail in 40 x of (A) showing a polymorphonuclear and macrophages infiltration. (C) - After 21 days were observed a mixed inflammatory infiltrate composed of macrophages and polymorphonuclear cells, with the thickness of the reactionary tissue uniform and a rudimentary organization. The connective tissue showed many basophilic formations, indicating the presence of dystrophic calcifications. (D) - Detail of (C) in 40 x. (E) - After 63 days were observed a mild mononuclear inflammatory infiltrate, predominantly macrophage and with eventual randomly dispersed polymorphonuclear cells. The thickness of the capsule formed was uniform. Many dystrophic calcifications have been found on the periphery of the reaction tissue

At 21 days, there was an increase in the degree of fibrous reaction tissue. The predominantly mononuclear inflammatory infiltrate varied from mild to moderate with occasional polymorphonuclear cells. In two specimens, dystrophic calcification was noted on the periphery of the reaction tissue.

At 63 days, the thickness of the reaction tissue was markedly thin and regular with few leukocytes infiltrating the structure. The collagen fiber formation, in some cases, was moderate. In two specimens, dystrophic calcification was observed on the periphery of the reaction tissue.

###  Zinc oxide-eugenol 

**Figure 4:**
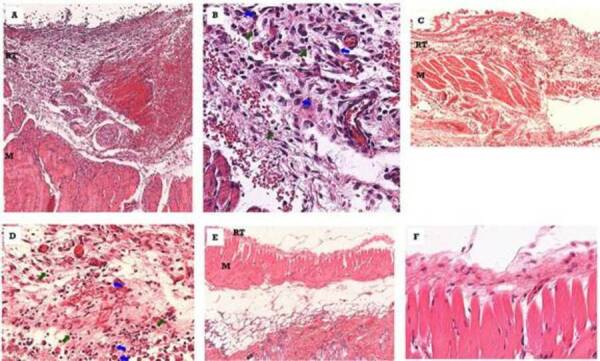
Representative photomicrographs of the tissue response observed in the subcutaneous connective tissue of mice after implantation of the zinc oxide-eugenol (ZOE) cylinder (10 × and 40 x magnifications) Blue arrow - PMN and Green arrow - macrophages. M - muscle, RT - reactional tissue. (A) - After 7 days were observed a reduced collagen density and edema was observed, with predominantly macrophage inflammatory infiltrate and with a marked presence of polymorphonuclear cells. (B) - Detail in 40 x of (A). (C) - At 21 days, were observed a mononuclear infiltrate predominantly macrophage, PMN were eventually observed. (D) - Detail in 40 x of (C). (E) - After 63 days, were observed a thin thickness of the reaction tissue and the reduced amount of inflammatory cells characterized by macrophages and polymorphonuclear cells.

Figure 4 is a representative image of the tissue response observed in the mice subcutaneous connective at 7 (A, B), 21 (C, D) and 63 (E, F) days after implantation of the Zinc Oxide-Eugenol cylinder (ZOE). In the 7-day period, a peripheral reaction tissue with reduced collagen density and edema was observed, with predominantly macrophage inflammatory infiltrate and with a marked presence of polymorphonuclear cells, especially of the neutrophil type, although occasionally eosinophils were observed. The thickness was uniform throughout the length of contact with the material.

At 21 days, the reaction tissue formed a very thin and uniform capsule in thickness, with slight fibrous tissue and a discrete mononuclear infiltrate predominantly macrophage. Neutrophils and eosinophils were eventually observed.

The main characteristic in the 63-day experimental period with regard to zinc oxide-eugenol was the thin thickness of the reaction tissue and the reduced amount of inflammatory cells characterized by macrophages and polymorphonuclear cells.

### Sham

**Figure 5:**
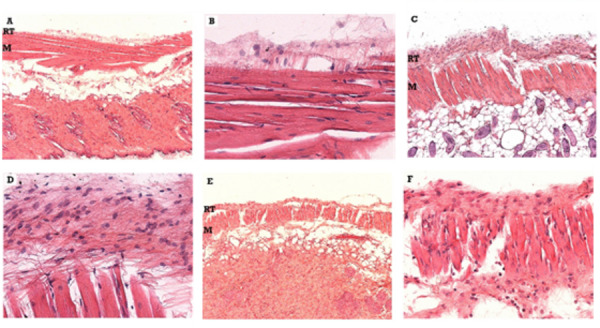
Representative photomicrographs of the tissue response observed in the subcutaneous connective tissue of mice after performing the experimental surgical procedure without implantation of the material (sham) (10 × and 40 x magnifications) Blue arrow - PMN and Green arrow - macrophages. M - muscle, RT - reactional tissue. (A) - After 7 days were observed a delicate tissue structure and no edema was observed. (B) - Detail in 40 x of (A). (C) - At 21 days, were observed a slight increase in the thickness of the reaction tissue, also no inflammatory infiltrate or edema. (D) - Detail in 40 x of (C). (E) - Afer 63 days, the reaction tissue in the area was thin, well-organized and discretely fibrous, with no edema or local infiltration by leukocytes.

Figure 5 is a representative image of the tissue response observed in the subcutaneous conjunctive of mice at 7 (A, B), 21 (C, D) and 63 (E, F) days after the surgical procedure (sham).

At 7 days, the connective tissue was markedly loose and infiltrated by eventual mononuclear leukocytes. The tissue structure was delicate and no edema was observed.

At 21 days, the Sham group revealed a slight increase in the thickness of the reaction tissue when compared to specimens from the 7-day period. There was no inflammatory infiltrate or edema.

At 63 days, the reaction tissue in the area was thin, well-organized and discretely fibrous, with no edema or local infiltration by leukocytes.

### Semi-quantitative Microscopic Analysis

**Figure 6:**
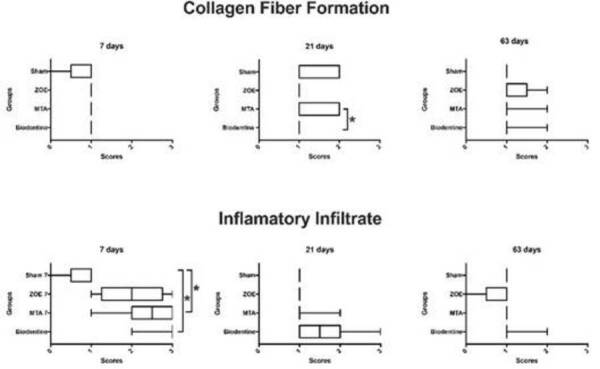
Graphical representation of the collagen fiber formation and inflammatory infiltrate scores around the implanted material, at 7, 21 and 63 days after implantation in the subcutaneous tissue of mice. Values expressed in micrometers for specimens from the Biodentine™, Mineral trioxide aggregate (MTA), Zinc oxide-eugenol (ZOE) groups and experimental surgical procedure without implantation (sham). * p <0.05

 Collagen Fiber Formation (  Figure 6  ) 

At 7 days, all groups (Biodentine™, Pro-Root MTA, ZOE and sham) induced a slight fibrous connective tissue around the implant (p> 0.05).

At 21 days, the fibrous became greater around the Pro-Root MTA and the specimens of the sham group. These experimental groups showed a statistically significant difference when compared to the Biodentine™ and ZOE groups (p <0.05), which remained with slight fibrosis.

At 63 days, all groups showed slight fibrosis in most specimens (p> 0.05).

###  Inflammatory infiltrate (Figure 6) 

At 7 days, Biodentine™, Pro-Root MTA and ZOE induced the recruitment of inflammatory cells of moderate to intense acuteness to the subcutaneous connective tissue around the implant, unlike the sham group that had a mild inflammatory infiltrate. This response was more intense for Biodentine™ and Pro-Root MTA, which showed a statistically significant difference when compared to the ZOE and sham group (p <0.05).

At 21 and 63 days, most specimens remained with a discrete infiltrate (p> 0.05).

### Quantitative Microscopic Analysis

###  Thickness of the fibrous capsule (  Figure 7 ) 

At 7 days, the thickness of the fibrous capsule was increased around the Biodentine™ and ZOE specimens, compared to the sham and Pro-Root MTA groups (p <0.05). In the periods of 21 and 63 days, the tissue around the materials did not increase in thickness and was similar to the sham group (p> 0.05).

**Figure 7:**
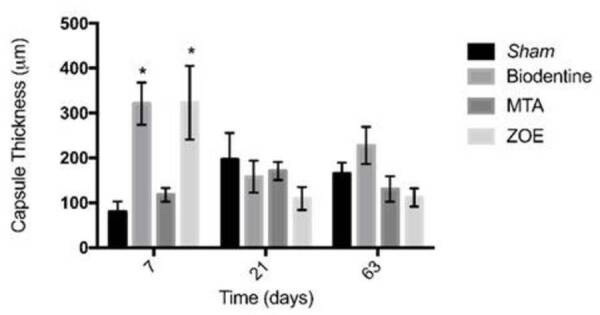
Graphical representation of the thickness of the fibrous capsule around the implanted material, at 7, 21 and 63 days after implantation in the subcutaneous tissue of mice. Values expressed in micrometers for specimens from the Biodentine™, Mineral trioxide aggregate (MTA), Zinc oxide-eugenol (ZOE) groups and experimental surgical procedure without implantation (sham). * p <0.05

**Figure 8:**
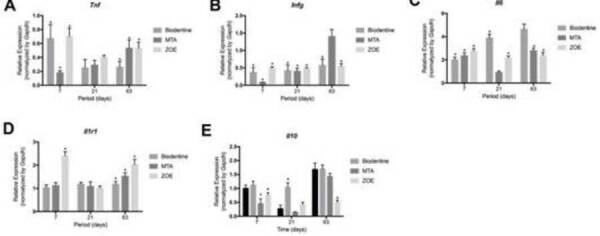
Graphical representation of the gene expression related to *Tnf* (A), *Infg* (B), *Il6* (C), *Il1r1* (D) e *Il10* (E) for specimens from the Biodentine™, Mineral trioxide aggregate (MTA), zinc oxide and eugenol (ZOE) groups and experimental surgical procedure without implantation (sham), at 7, 21 and 63 days after implantation in the subcutaneous tissue of mice. * p <0.05 compared to the sham group.

*Evaluation of Gene Expression (* Figure 8  ) 

###  Tnf 

At 7 days, Biodentine™, Pro-Root MTA and ZOE inhibited Tnf expression (p <0.05), but showed no effect at 21 days (p> 0.05). At 63 days, again Biodentine™, Pro-Root MTA and ZOE inhibited Tnf expression (p <0.05). The greatest inhibition of gene expression occurred in subcutaneous connective tissue in contact with Pro-Root MTA, at 7 days, and with Biodentine™ at 63 days.

###  Infg 

At 7 and 21 days, Biodentine™, Pro-Root MTA and ZOE inhibited Infg expression (p <0.05). This inhibition was maintained until 63 days for Biodentine™ and ZOE (p <0.05), but it was not modulated by the Pro-Root MTA in that period (p> 0.05). The greatest inhibition occurred in contact with Pro-Root MTA at 7 days and with Biodentine™ and ZOE at 63 days, with no statistical difference between them (p> 0.05).

###  Il6 

At 7 days, Biodentine™, Pro-Root MTA and ZOE induced the expression of Il6 (p <0.05). This induction was maintained until 21 days for Biodentine™ and ZOE (p <0.05), but it was not modulated by Pro-Root MTA in that period (p> 0.05). At 63 days, Pro-Root MTA and ZOE inhibited Il6 while Biodentine™ had no effect on gene modulation (p> 0.05).

###  Il1r1 

At 7 days, ZOE induced the expression of Il1r1 (p <0.05) while Biodentine™ and Pro-Root MTA had no effect (p> 0.05). At 21 days, no material modulated gene expression (p> 0.05), unlike the 63-day period, when Biodentine™, Pro-Root MTA and ZOE induced Il1r1 (p <0.05).

###  Il10 

At 7 days, Pro-Root MTA and ZOE inhibited the expression of Il10 (p <0.05) and Biodentine™ had no effect (p> 0.05). At 21 days, Pro-Root MTA and ZOE had no effect (p> 0.05) while Biodentine™ induced Il10 (p <0.05). At 63 days, ZOE inhibited Il10 (p <0.05), while Pro-Root MTA and Biodentine™ had no effect (p> 0.05).

### Summary of the outcomes observed in each group

###  Biodentine 

At 7 days, this group presented a disorganized and thicker fibrous membrane, inflammatory cells (neutrophils and macrophages), punctual dystrophic calcifications, inhibition of TNF-α, INF-γ and increased expression of IL-6.

At 21 days, the findings were slight fibrous tissue, with rudimentary organizational structure, mild to moderate mixed inflammatory infiltrate, presence of dystrophic calcifications, inhibition of INF-γ, continued increased expression of IL-6 and IL-10.

At 63 days, it was observed a uniform and slight fibrous capsule, mild mononuclear inflammatory infiltrate, many dystrophic calcifications, significant inhibition of TNF-α, INF-γ, continued increased expression of IL-6, slight increased expression of IL1r1 and continued increased expression of IL-10 (but similar to the sham).

###  Pro-Root MTA 

At 7 days, this group presented a uniform tissue reaction with slight fibrous, a predominantly macrophage inflammatory infiltrate, significant inhibition of TNF-α, INF-γ, and IL-10 and increased expression of IL-6.

At 21 days, there were an increase in the degree of fibrous reaction tissue, mild to moderate predominantly mononuclear inflammatory infiltrate, inhibition of INF-γ and reduced expression of IL-6 and IL-10, similar to sham.

For 63 days, this group showed a thin reaction tissue, with collagen fiber formation and few leukocytes, inhibition of TNF-α and IL-6, increased expression of INF-γ and IL-10 (but similar to the sham) and increased expression of IL1r1.

###  Zinc oxide-eugenol 

At 7 days, this group presented a uniform and thicker fibrous tissue, with edema, reduced collagen density and inflammatory cells (neutrophils and macrophages), inhibition of TNF-α, INF-γ and IL-10, and considerable increased expression of IL-6 and IL1r1.

At 21 days, it was observed a uniform, thin and slight fibrous capsule, few macrophages, inhibition of INF-γ, increased expression of IL-6 and reduced expression of IL-10, similar to sham.

At 63 days, the findings were a thin reaction tissue, reduced amount of inflammatory cells, inhibition expression of TNF-α, IL-6, being significant for INF-γ and IL-10 and increased expression of IL1r1.

## Discussion

The null hypothesis of the study should be rejected since some immune-inflammatory reactions were different between MTA and Biodentine.

To be used directly in pulp tissue, any dental material must have adequate physical and mechanical properties and satisfactory biological properties. Thus, tissue compatibility tests are essential ^21^ and for that several methodologies can be used, among them, the evaluation of the *in vivo* tissue subcutaneous response of isogenic mice stands out ^20^. This method allows a detailed assessment of the characteristics of the reaction tissue and provides sufficient information about inflammatory and immune responses, elucidating the mechanisms of action of the evaluated materials.

Experimental periods of 7, 21, and 63 days were selected based on previous studiy from our research group that evaluated tissue response after subcutaneous implantation of different materials ^20^. According to International Organization for Standardization (number 10993-6:2007), where there is no or minimal degradation, local tissue responses shall be evaluated at 1 week to 12 weeks after implantation. During the first two weeks after implantation, the reaction due to the surgical procedure itself may be difficult to distinguish from the tissue reaction evoked by the implant. In muscle and connective tissues, depending on the species, and the severity of the surgical trauma, a steady state is seen in the cell population after 9 weeks to 12 weeks (ISO 10993-6: 2007). Then, the periods of 7, 21 and 63 days comprise an initial acute response, an intermediate period without interference from the surgical procedure and a long-term chronic response to the materials. In addition, beyond the tissue response to the associated trauma of surgery, the local biological reaction depends on the properties of the materials and the test period shall be determined by the likely clinical exposure time, be continued until, or beyond a steady state has been reached with respect to the biological response. Considering that reparative dentinogenesis is a complex defense and healing pulp response, involving genes expression, signaling pathways activation and tissue mineralization and that both materials will remain reacting with the pulp tissue for a long period, the time points selected are justified. For a material to be considered suitable from a biological point of view, its toxicity must be low or zero. Tissue compatibility of MTA and Biodentine was confirmed by the present results, as supported by literature. Accordingly, the absence of cytotoxic effects is demonstrated in stem cells and odontoblasts ^22^ and these biomaterials were well tolerated by the subcutaneous connective tissues in the 60-day evaluation period ^23^.

As for inflammation, it is known that it is an essential process for tissue repair, and is even considered as the initial stage of repair, along with coagulation, followed by resolution of the inflammation and ended by revascularization ^24^. Thus, it is acceptable for a biocompatible material to trigger an inflammatory response in the initial evaluation periods ^25^, but this must be a controlled event and of mild magnitude. The results of the present study for ProRoot MTA and Biodentine^®^ are consistent with recent findings that showed similar moderate inflammatory tissue response on day 7 and that inflammatory cell numbers decreased over time for both biomaterials ^23^.

The recognition of an offending agent triggers the production of pro-inflammatory cytokines and chemokines capable of attracting inflammatory cells to the region, such as neutrophils and macrophages ^21^ ^,^ ^26^, and to stimulate the migration of undifferentiated pulp cells to the pulp lesion site ^27^, being able to differentiate into odontoblasts, thus forming a restorative barrier to dentin ^28^. Thus, the study of inflammatory markers such as cytokines and interleukins are extremely important for understanding the tissue repair process of the dental pulp. They are modulators of immune and inflammatory responses, and can play a pro- or anti-inflammatory role, or both, depending on the cells and tissues analyzed ^29^, and can favor the worsening of inflammation or the repair process.

In agreement with the present findings, previous studies have shown that Biodentine™ is capable of promoting an initial inflammatory reaction, which becomes discreet in a short time ^30^. The fibrous capsule is an important inflammatory marker ^31^, as the materials can release proinflammatory substances, which stimulate the maintenance of the capsule as an attempt of the body to isolate that material ^32^. In the present study, it was observed that Biodentine™ initially stimulated the formation of a thicker fibrous capsule than Pro-Root MTA and the sham group, but it was not statistically different from the group induced by zinc oxide-eugenol. However, with time, this thickness decreased and it was no longer possible to observe a difference with the other experimental groups.

Initially, Biodentine promoted a more intense reaction in comparison with MTA, showing a tendency for higher expression of TNF-α, INF-γ and IL-10. Except for Il1r1, gene expression induced by Biodentine had a certain similarity to that induced by ZOE, mainly with regard to neutrophil and macrophages activating cytokines (TNF-α and INF-γ), confirming the initial stimulatory effect. This result correlates with histological analysis that evidenced a great infiltration of inflammatory cells at 7 days. With the presence of macrophages and the release of chemical mediators, migration and activation of fibroblasts is intensified, which may explain in part the thicker fibrous membrane observed in the Biodentine group. In parallel, expression of IL-10 was similar to the sham group and higher than MTA and ZOE, indicating that the initial response to Biodentine envolves a protective function. The expression of IL-10 remained increased at 21 and 63 days.

Interleukin-6 is an activator of the immune system, playing a pivotal role during the transition from acute to chronic inflammation and acquired immunity ^33^. Although similar to MTA at 7 days, expression of IL-6 was higher in relation to sham group, corroborating the initial intense reaction promoted by Biodentine.

Regarding MTA, a study by Cintra et al. ^34^ found that at 7 days, MTA induced an inflammatory response ranging from mild to moderate, which did not persist over time. Da Foncesca et al. ^30^ evaluated the inflammatory process caused by MTA and Biodentine™, by measuring the number of inflammatory cells present in the connective tissue of rats, in sections stained with HE, and the numerical density of cells immunized with *Il6*. An inflammatory reaction initially induced by Biodentine™ was observed, compared to MTA. After 15 days, there was a significant reduction in the number of inflammatory cells and immunoexpression of *Il6* for both groups. According to Giraud et al. ^35^, Biodentine™ provided less expression of Il6 throughout the inflammatory process. In the present study, both Biodentine™ and Pro-Root MTA were able to stimulate *Il6* expression in the initial periods. However, we observed that in the last evaluated period of 63 days, Pro-Root MTA inhibited the expression of this cytokine, which did not happen with Biodentine™, partially disagreeing with previous reports. Interleukin (IL)-6 has different biological effects and can act as a mediator of the host's response after injury and tissue infection, especially because it has activity in the regulation of adhesion molecules, inducing angiogenesis and increasing vascular permeability and inflammatory edema ^36^.

Similar result in IL-6 expression occurred only for MTA at 21 and 63 days, while Biodentine remained this expression increased in all experimental periods, being higher than MTA, mainly at 21 days.

The intermediate period of 21 days did not show relevant findings for pro-inflammatory markers differing both materials (*TNF-α, INF-γ* and *Il1r1*). On the other hand, expression of IL-6 and IL-10 was markedly higher for Biodentine, in comparison with MTA.

According to the present findings, it can be inferred that MTA plays a mechanism that inhibits the inflammatory response, since this material evidenced a significant reduction in the expression of the proinflammatory markers *Tnf* and *Infg* at 7 days, although without statistical difference from Biodentine*.* On the other hand, the inhibition of *Il-10* expression in the initial periods by MTA can demonstrate an indirectly pro-inflammatory response that stimulates tissue repair ^37^.

Gamma interferon (IFN-γ) is a cytokine that has important inflammatory properties, acting as a trigger for macrophages to produce and release inflammatory mediators such as reactive oxygen species ^36^. In 2013, Elsalhy and collaborators ^36^ observed high levels of IFN-γ in the pulps of teeth affected with caries lesions, as well as pulps that presented irreversible pulpitis, thus presenting their immunomodulatory and inflammatory effects.

At final period (63 days), the inhibition of IFN-γ was kept only for Biodentine, while MTA had a higher expression similarly to the sham group, suggesting a participation of macrophages and their mediators in tissue repair promoted by MTA at late stages, likely related to fibrous formation.

The IL‐10 is considered an antiinflammatory cytokine, being the main inhibitor of proinflammatory mediator synthesis and macrophage activity ^35^ ^,^ ^38^. Its expression was elevated for MTA and Biodentine at 63 days, demonstrating that both biomaterials are able to modulate the inflammatory process and to stimulate tissue repair, corroborating previous evidences ^21^ ^,^ ^23^.

Interestingly, the materials increased the expression for Il1r1 at the final stage, while this marker did not show relevant changes at 7 and 21 days in response to MTA and Biodentine. This high expression can be explained by the recognition of a foreign material by the tissue defense system during the repair process. IL-1 receptor (Il1r1) mediates almost all IL-1 actions ^39^, being found in a large variety of cells of innate immune system as well as selective T cell populations in the adaptive immune system. Evidence indicates that IL-1 family members play role in innate and adaptive lymphoid cell differentiation and function ^40^. It has been reported that lymphoid cells may act limiting inflammation and supporting tissue repair and wound healing mechanisms during the resolution phase of immune responses against certain pathogens and insults ^41^. Then, our findings of stimulated expression of Il1r1 at the cronic stage can be suppoted by this evidence.

When properly indicated, cases treated with MTA and Biodentine™ can achieve a success rate of 84.6% and 92.3%, respectively ^42^. This success rate may be associated with the mechanism of action of both materials, being considered bioactives. Calcium hydroxide is the common product resulting from the tissue reaction, which has two important physiological properties: 1- release of calcium ions, fundamental for cell adhesion and proliferation; and 2- alkalinization of the medium, generating an antibacterial environment, modulating the production of cytokines, inducing differentiation and migration of cells producing dentinal and bone-dentin tissue, and stimulating the formation of dentin bridge ^43^. Consistent with the literature ^44^, the mineralizing potential of Biodentine™ and Pro-Root MTA was confirmed in the present study, after microscopic evidence of many basophilic formations, compatible with dystrophic calcifications, in the most peripheral portion of the connective tissue. In agreement with our findings, Karabulut et al. ^23^ evidenced the presence of dystrophic calcification in the connective tissue adjacent to the Biodentine^®^ and ProRoot MTA. The bioactivity of both materials was also confirmed by alkaline pH, capacity to release calcium ions and precipitation of apatite crystalline structures over cement and dentin substrates ^45^. These results together provide a solid foundation for the capacity of the Biodentine^®^ and ProRoot MTA to induce the formation of high-quality mineralized tissue for regeneration of the dentin-pulp complex.

In general, the results of the present study allow elucidating the repair inducing mechanisms stimulated by Biodentine™ and Pro-Root MTA. Knowledge gained from studying the cytokine interactions and influences provides a better understanding of the complex orchestrated events involded in dentin bridge formation by both biomaterials. However, direct extrapolations to clinical conditions must be exercised with caution because of obvious limitations of preclinical animal studies. Although these materials have been previous studied, new researchers must be performed regarding the mechanism of Biodentine™️ and Pro-Root MTA during the repair process. More precise molecular biology techniques could be adopted in order to evaluate molecular aspects. These studies would favor the improvement of these materials, and may be increase the effectiveness and specificity.

According to the results obtained, it was concluded that, in general, the evaluated materials, Pro-Root MTA and Biodentine™, showed tissue compatibility, mediated inflammation, with increased fibrous tissue and production of pro- and anti-inflammatory cytokines.
